# Complete genome sequence of *Meiothermus silvanus* type strain (VI-R2^T^)

**DOI:** 10.4056/sigs.1042812

**Published:** 2010-07-29

**Authors:** Johannes Sikorski, Brian J Tindall, Stephen Lowry, Susan Lucas, Matt Nolan, Alex Copeland, Tijana Glavina Del Rio, Hope Tice, Jan-Fang Cheng, Cliff Han, Sam Pitluck, Konstantinos Liolios, Natalia Ivanova, Konstantinos Mavromatis, Natalia Mikhailova, Amrita Pati, Lynne Goodwin, Amy Chen, Krishna Palaniappan, Miriam Land, Loren Hauser, Yun-Juan Chang, Cynthia D. Jeffries, Manfred Rohde, Markus Göker, Tanja Woyke, James Bristow, Jonathan A. Eisen, Victor Markowitz, Philip Hugenholtz, Nikos C. Kyrpides, Hans-Peter Klenk, Alla Lapidus

**Affiliations:** 1 DSMZ - German Collection of Microorganisms and Cell Cultures GmbH, Braunschweig, Germany; 2 DOE Joint Genome Institute, Walnut Creek, California, USA; 3 Los Alamos National Laboratory, Bioscience Division, Los Alamos, New Mexico, USA; 4 Biological Data Management and Technology Center, Lawrence Berkeley National Laboratory, Berkeley, California, USA; 5 Oak Ridge National Laboratory, Oak Ridge, Tennessee, USA; 6 HZI – Helmholtz Centre for Infection Research, Braunschweig, Germany; 7 University of California Davis Genome Center, Davis, California, USA

**Keywords:** thermophilic, aerobic, biofouler, colored biofilm in paper industry, Gram-negative, *Thermales*, *Deinococci*, GEBA

## Abstract

*Meiothermus silvanus* (Tenreiro *et al.* 1995) Nobre *et al*. 1996 belongs to a thermophilic genus whose members share relatively low degrees of 16S rRNA gene sequence similarity. *Meiothermus* constitutes an evolutionary lineage separate from members of the genus *Thermus*, from which they can generally be distinguished by their slightly lower temperature optima. *M. silvanus* is of special interest as it causes colored biofilms in the paper making industry and may thus be of economic importance as a biofouler. This is the second completed genome sequence of a member of the genus *Meiothermus* and only the third genome sequence to be published from a member of the family *Thermaceae*. The 3,721,669 bp long genome with its 3,667 protein-coding and 55 RNA genes is a part of the *** G****enomic* *** E****ncyclopedia of* *** B****acteria and* *** A****rchaea * project.

## Introduction

Strain VI-R2^T^ (= DSM 9946) was first described as ‘*Thermus silvanus*’ by Tenreiro *et al*. in 1995 [1]. One year later it was formally named and transferred from the genus *Thermus* into the then novel genus *Meiothermus* by Nobre *et al*. [2]. Currently, there are nine species within the genus *Meiothermus* [3,4]. The genus name derives from the Greek words ‘meion’ and ‘thermos’ meaning ‘lesser’ and ‘hot’ to indicate an organism in a less hot place [2,3]. The species name was given in honor of Manuel T. Silva, a Portuguese microbiologist and immunologist [1]. Strain VI-R2^T^ was isolated from the hot spring (vent temperature, 56°C; pH 8.9) located at the end of a 450 m tunnel and from thermal water (temperature 33°C; pH 8.8) piped to a spa at Vizela in northern Portugal [1].

Members of the genus *Meiothermus* have been isolated from natural hot springs and artificial thermal environments [2,5] in Russia [6], Central France [7], Northern and Central Portugal [1,8], North-Eastern China [9], Northern Taiwan [10], Iceland [11] and the Azores [4]. Interestingly, the genus *Meiothermus* is heterogeneous with respect to pigmentation. The yellow pigmented species also form a distinct group on the basis of the 16S rRNA gene sequence similarity, whereas the red/orange pigmented strains form two groups, one comprising *M. silvanus* and the other the remaining species [8,9]. Like all members of the class *Deinococci,* the lipid composition of the cell membrane of members of the genus *Meiothermus* contains unusual and characteristic structures.

*M. silvanus* is well known to form colored biofilms in the paper industry, which makes this species an economic threat [12,13]. *M. silvanus* uses thread-like organelles for adhesion and biofilm formation to grow on stainless steel [14]. However, coating of stainless steel with diamond-like carbon or certain fluoropolymers reduced or almost eliminated adhesion and biofilm growth of *M. silvanus* [14]. Other strategies to combat *M. silvanus* in the paper industry include electrochemical inactivation (oxidation) using different levels of chloride concentration [15]. Here, the inactivation was mainly due to the electrochemically generated chlorine/hypochlorite [15]. A patent based on different natural plant extracts inhibiting biofilm formation of thermophilic species in paper or board machines, amongst them *M. silvanus*, has been recently issued [16].

The 16S rRNA genes of the seven other type strains in the genus *Meiothermus* share between 88.5% (*Meiothermus chliarophilus* [1]) and 89.8% (*Meiothermus cerbereus* [11]) sequence identity with strain VI-R2^T^, whereas the other type strains from the family *Thermaceae* share 85.8 to 87.8% sequence identity [17]. In addition to being found on  paper and board machines [12] uncultured clone 16S rRNA gene sequences very similar to *M. silvanus* RI-V2^T^ (X84211) have also been detected in the gut of an invasive wood-boring beetle (98% identity, EU148672) [18] and in seawater adjacent to a *Pacillopora meandrina* coral colony at Palmyra Atoll (99% identity, EU249942). Environmental samples and metagenomic surveys do not surpass 84% sequence similarity to the 16S rRNA gene sequence of strain RI-V2^T^ (status May 2010). Here we present a summary classification and a set of features for *M. silvanus* RI-V2^T^, together with the description of the complete genomic sequencing and annotation.

## Classification and features

**Figure 1:**
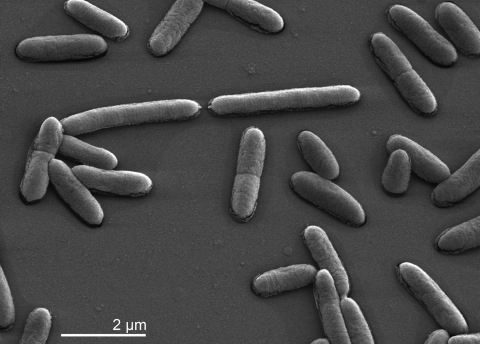
Scanning electron micrograph of *M. silvanus* VI-R2^T^.

A physiological description based on five strains of the species is given by Tenreiro *et al.* [1]. The cells are described as Gram-negative nonmotile rods with variable lengths and 0.5 to 0.8 µm in width (Table 1 and Figure 1). On *Thermus* medium colonies are orange-red pigmented and 0.5 to 1.2 µm in diameter after 72 h of growth [1]. The optimum pH is between 8.0 and 8.5; growth does not occur at pH 5.0 or 10.0. Yeast extract is required for growth [1]. All strains are oxidase positive and catalase negative. Nitrate is reduced to nitrite. Strain VI-R2^T^ is negative for the enzyme α-galactosidase but positive for β-galactosidase. Casein, elastin, gelatin, hide powder azure, and starch are degraded. The hydrolysis of fibrin is weak or negative. Strain VI-R2^T^ utilizes D-glucose, D-fructose, D-mannose, D-galactose, D-xylose, maltose, lactose, D-melibiose, glycerol, D-mannitol, D-sorbitol, ribitol, pyruvate, L-glutamate, L-asparagine, L-serine, L-glutamine, and L-proline [1]. Strain VI-R2^T^ does not utilize L-arabinose, L-rhamnose, sucrose, D-cellobiose, D-trehalose, D-raffinose, meso-erythritol, galactitol, *myo*-inositol, acetate, succinate, citrate, salicin, or acetamide [1]. Further metabolic traits are listed elsewhere [7]. Also, strain VI-R2^T^ produces polysaccharide inclusions [1].

**Table 1:** Classification and general features of *M. silvanus* VI-R2^T^ according to the MIGS recommendations [19]

**MIGS ID**	**Property**	**Term**	**Evidence code**
	Current classification	Domain *Bacteria*	TAS [20]
		Phylum ‘*Deinococcus* -*Thermus*	TAS [21,22]
		Class *Deinococci*	TAS [23-25]
		Order *Thermales*	TAS [26,27]
		Family *Thermaceae*	TAS [24,27]
		Genus *Meiothermus*	TAS [2,7]
		Species *Meiothermus silvanus*	TAS [1,6]
		Type strain RI-V2	TAS [6]
	Gram stain	negative	TAS [1]
	Cell shape	rod	TAS [1]
	Motility	nonmotile	TAS [1]
	Sporulation	no	TAS [1]
	Temperature range	40°C–65°C	TAS [1]
	Optimum temperature	55°C	TAS [1]
	Salinity	does not grow with 1% or more NaCl	TAS [1]
MIGS-22	Oxygen requirement	aerobic	TAS [1]
	Carbon source	a diverse set of sugars	TAS [1]
	Energy source	carbohydrates	TAS [1]
MIGS-6	Habitat	hot springs	TAS [1]
MIGS-15	Biotic relationship	free-living	TAS [1]
MIGS-14	Pathogenicity	not reported	
	Biosafety level	1	TAS [28]
	Isolation	hot spring	TAS [1]
MIGS-4	Geographic location	Vizela, Portugal	TAS [1]
MIGS-5	Sample collection time	1995 or before	TAS [1]
MIGS-4.1 MIGS-4.2	Latitude Longitude	41.38 8.32	NAS
MIGS-4.3	Depth	unknown	
MIGS-4.4	Altitude	157 m	NAS

Evidence codes - IDA: Inferred from Direct Assay (first time in publication); TAS: Traceable Author Statement (i.e., a direct report exists in the literature); NAS: Non-traceable Author Statement (i.e., not directly observed for the living, isolated sample, but based on a generally accepted property for the species, or anecdotal evidence). These evidence codes are from of the Gene Ontology project [29]. If the evidence code is IDA, then the property was directly observed by one of the authors or an expert mentioned in the acknowledgements.

**Figure 2:**
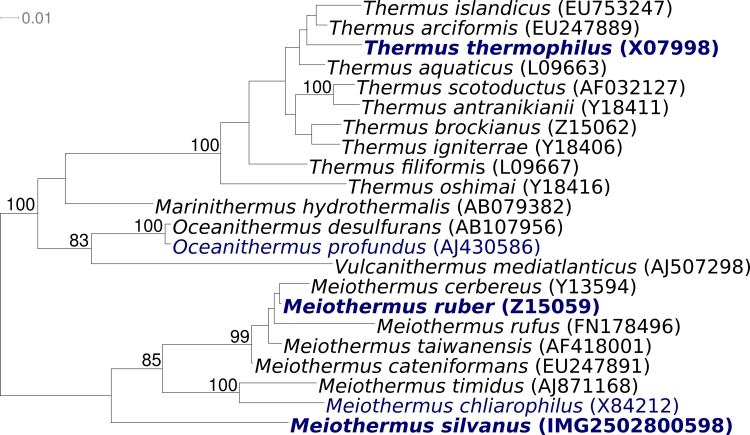
Phylogenetic tree highlighting the position of *M. silvanus* VI-R2^T^ relative to the type strains of the other species within the genus and to the other type strains within the family *Thermaceae*. The tree was inferred from 1,442 aligned characters [30,31] of the 16S rRNA gene sequence under the maximum likelihood criterion [32] and rooted in accordance with the current taxonomy [33]. The branches are scaled in terms of the expected number of substitutions per site. Numbers above branches are support values from 900 bootstrap replicates [34] if larger than 60%. Lineages with type strain genome sequencing projects registered in GOLD [35] are shown in blue, published genomes in bold, i.e. *Thermus thermophilus* (AP008226) and the type species of the genus, *M. ruber* [36].

Figure 2 shows the phylogenetic neighborhood of *M. silvanus* VI-R2^T^ in a 16S rRNA-based tree. The sequences of the two 16S rRNA gene copies in the genome of *M. silvanus* VI-R2^T^ do not differ from each other, but differ by six nucleotides from the previously published 16S rRNA sequence from DSM 9946 (X84211).

### Chemotaxonomy

Thin-layer chromatography of the polar lipids from *M. silvanus* revealed a single phospholipid (PL-2) and two prominent glycolipids GL-la and GL-lb [37]. Although the structure of the major phospholipid has not been investigated from *M. silvanus* it has the same Rf value as the 2’-O-(1, 2-diacyl-sn-glycero-3-phospho) –3’-O-(α-N-acetyl-glucosaminyl)-N-glyceroyl alkylamine from *M. ruber* [38]. The glycolipids are derivatives of a Glcp-> Galp-> GalNAcyl-> Glcp-> diacyl glycerol [37].

Based on mass spectral data it appears that there may be three distinct derivatives, differing in the fatty acid amide linked to the galactosamine [37]. These may be divided into one compound containing exclusively 2-hydroxylated fatty acids (mainly 2-OH iso-17:0) and a mixture of two compounds that cannot be fully resolved by thin layer chromatography, carrying either 3-hydroxylated fatty acids or unsubstituted fatty acids. The basic glycolipid structure dihexosyl – N-acyl-hexosaminyl – hexosyl – diacylglycerol is a feature common to all members of the genera *Thermus* and *Meiothermus* examined to date. There is currently no evidence that members of the family *Thermaceae* (as currently defined) produce significant amounts of polar lipids containing only two aliphatic side chains. The consequences of having polar lipids containing three aliphatic side chains on membrane structure has yet to be examined. Such peculiarities also indicate the value of membrane composition in helping to unravel evolution at a cellular level [36]. The major fatty acids of the total polar lipids are anteiso-C_15:0_ (22.4%), iso-C_15:0_ (16.8%) and iso-C_18:0_ (12.2%), followed by iso-C_17:0_-2OH (10.5%) and iso-C_17:0_ and anteiso-C_17:0_ (each 8.5%) [37]. The glycolipid GL-la is characterized by a large amount of the fatty acid iso-C_17:0_-2OH (19.2%), which is nearly completely absent from GL-lb and the phospholipid PL-2 [37]. Menaquinone 8 was the only respiratory lipoquinone detected in all strains [1]. The structure of the red pigment has not been characterized in contrast to that of *M. ruber* [39].

## Genome sequencing and annotation

### Genome project history

This organism was selected for sequencing on the basis of its phylogenetic position [40], and is part of the *** G****enomic* *** E****ncyclopedia of* *** B****acteria and* *** A****rchaea * project [41]. The genome project is deposited in the Genome OnLine Database [35] and the complete genome sequence is deposited in GenBank. Sequencing, finishing and annotation were performed by the DOE Joint Genome Institute (JGI). A summary of the project information is shown in Table 2.

**Table 2:** Genome sequencing project information

**MIGS ID**	**Property**	**Term**
MIGS-31	Finishing quality	Finished
MIGS-28	Libraries used	Three genomic libraries: Sanger 8 kb pMCL200 and fosmid libraries, one 454 pyrosequence standard library
MIGS-29	Sequencing platforms	ABI3730, 454 Titanium, Illumina GAii
MIGS-31.2	Sequencing coverage	8.3× Sanger; 16.6× pyrosequence
MIGS-30	Assemblers	Newbler version 1.1.02.15, Arachne
MIGS-32	Gene calling method	Prodigal 1.4, GenePRIMP
	INSDC ID	CP002042 chromosome CP002043 plasmid pMESIL01 CP002044 plasmid pMESIL02
	Genbank Date of Release	June 4, 2010
	GOLD ID	Gc01327
	NCBI project ID	29551
	Database: IMG-GEBA	2502790002
MIGS-13	Source material identifier	DSM 9946
	Project relevance	Tree of Life, GEBA

### Growth conditions and DNA isolation

*M. silvanus* VI-R2^T^, DSM 9946, was grown in DSMZ medium 86 (Castenholz Medium) [42] at 50°C. DNA was isolated from 0.5-1 g of cell paste using Qiagen Genomic 500 DNA Kit (Qiagen, Hilden, Germany) following the standard protocol as recommended by the manufacturer, with modification st/LALMP as described in Wu *et al.* [41].

### Genome sequencing and assembly

The genome was sequenced using a combination of Sanger and 454 sequencing platforms. All general aspects of library construction and sequencing can be found at the JGI website (http://www.jgi.doe.gov/). Pyrosequencing reads were assembled using the Newbler assembler version 1.1.02.15 (Roche). Large Newbler contigs were broken into 3,908 overlapping fragments of 1,000 bp and entered into assembly as pseudo-reads. The sequences were assigned quality scores based on Newbler consensus q-scores with modifications to account for overlap redundancy and adjust inflated q-scores. A hybrid 454/Sanger assembly was made using the Arachne assembler. Possible misassemblies were corrected and gaps between contigs were closed editing in Consed, custom primer walks from sub-clones or PCR products. A total of 323 Sanger finishing reads were produced to close gaps, to resolve repetitive regions, and to raise the quality of the finished sequence. 9,068,515 Illumina reads were used to improve the final consensus quality using an in-house developed tool (the Polisher) [43]. The error rate of the completed genome sequence is less than 1 in 100,000. Together, the combination of the Sanger and 454 sequencing platforms provided 26.9× coverage of the genome. The final assembly contains 42,181 Sanger reads and 335,557 pyrosequencing reads.

### Genome annotation

Genes were identified using Prodigal [44] as part of the Oak Ridge National Laboratory genome annotation pipeline, followed by a round of manual curation using the JGI GenePRIMP pipeline [45]. The predicted CDSs were translated and used to search the National Center for Biotechnology Information (NCBI) nonredundant database, UniProt, TIGRFam, Pfam, PRIAM, KEGG, COG, and InterPro databases. Additional gene prediction analysis and functional annotation was performed within the Integrated Microbial Genomes - Expert Review (IMG-ER) platform [46].

## Genome properties

**Figure 3:**
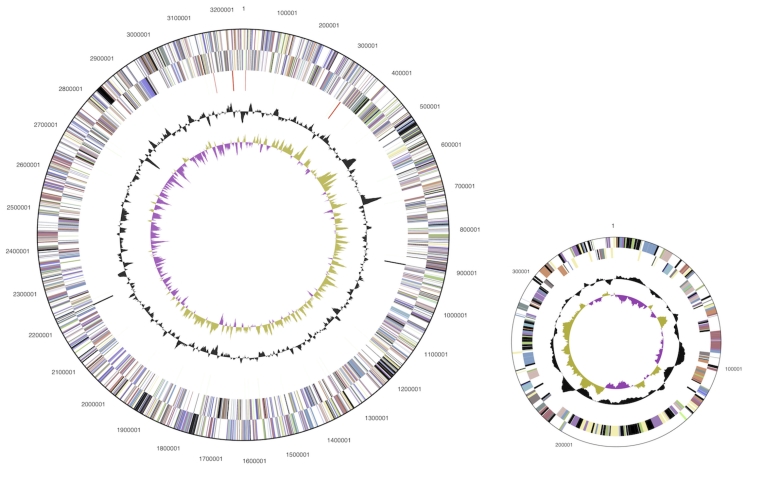
Graphical circular map of the genome and the larger of the two plasmids (not drawn to scale). From outside to the center: Genes on forward strand (color by COG categories), Genes on reverse strand (color by COG categories), RNA genes (tRNAs green, rRNAs red, other RNAs black), GC content, GC skew.

The genome consists of a 3,249,394 bp long chromosome, and two plasmids of 347,854 bp and 124,421 bp lengths, respectively, with a total G+C content of 62.7% (Table 3 and Figure 3). Of the 3,722 genes predicted, 3,667 were protein-coding genes, and 55 RNAs; 158 pseudogenes were also identified. The majority of the protein-coding genes (64.5%) were assigned a putative function while the remaining ones were annotated as hypothetical proteins. The distribution of genes into COGs functional categories is presented in Table 4.

**Table 3:** Genome Statistics

**Attribute**	**Value**	**% of Total**
Genome size (bp)	3,721,669	100.00%
DNA Coding region (bp)	3,283,226	88.22%
DNA G+C content (bp)	2,334,056	62.72%
Number of replicons	3	
Extrachromosomal elements	2	
Total genes	3,722	100.00%
RNA genes	55	1.48%
rRNA operons	2	
Protein-coding genes	3,667	98.52%
Pseudo genes	158	4.25%
Genes with function prediction	2,400	64.48%
Genes in paralog clusters	740	19.89%
Genes assigned to COGs	2,530	67.97%
Genes assigned Pfam domains	2,797	75.15%
Genes with signal peptides	1,249	33.56%
Genes with transmembrane helices	796	21.39%
CRISPR repeats	16	

**Table 4:** Number of genes associated with the general COG functional categories

**Code**	**value**	**%age**	**Description**
J	151	5.4	Translation, ribosomal structure and biogenesis
A	0	0.0	RNA processing and modification
K	166	6.0	Transcription
L	195	7.0	Replication, recombination and repair
B	2	0.1	Chromatin structure and dynamics
D	33	1.2	Cell cycle control, cell division, chromosome partitioning
Y	0	0.0	Nuclear structure
V	43	1.6	Defense mechanisms
T	120	4.3	Signal transduction mechanisms
M	128	4.6	Cell wall/membrane/envelope biogenesis
N	26	0.9	Cell motility
Z	0	0.0	Cytoskeleton
W	0	0.0	Extracellular structures
U	53	1.9	Intracellular trafficking, secretion, and vesicular transport
O	107	5.5	Posttranslational modification, protein turnover, chaperones
C	168	6.1	Energy production and conversion
G	218	7.9	Carbohydrate transport and metabolism
E	292	10.5	Amino acid transport and metabolism
F	85	3.1	Nucleotide transport and metabolism
H	110	4.0	Coenzyme transport and metabolism
I	88	3.2	Lipid transport and metabolism
P	152	5.5	Inorganic ion transport and metabolism
Q	52	1.9	Secondary metabolites biosynthesis, transport and catabolism
R	362	13.0	General function prediction only
S	227	8.2	Function unknown
-	1,192	32.0	Not in COGs
